# All together now: Geographically coordinated miticide treatment benefits honey bee health

**DOI:** 10.1111/1365-2664.14367

**Published:** 2023-01-26

**Authors:** Luke Woodford, Graeme Sharpe, Fiona Highet, David J. Evans

**Affiliations:** ^1^ Biomedical Sciences Research Complex University of St. Andrews St. Andrews UK; ^2^ Scotland's Rural College Ayrshire UK; ^3^ Science and Advice for Scottish Agriculture Edinburgh UK

**Keywords:** coordinated treatment, deformed wing virus, honey bee, landscape, miticide, *Varroa destructor*, vector, virus

## Abstract

1. Deformed wing virus (DWV) is a pathogenic virus of honey bees transmitted by the ectoparasitic mite *Varroa destructor*. Annual overwintering colony losses, accounting for ~25% of all colonies, are associated with high levels of Varroa‐DWV infestation. Effective miticide treatments are available to control Varroa. However, the absence of coordinated treatment means environmental transmission of mites continues unchecked. We aimed to determine whether rational, coordinated treatment is beneficial, and characterized the DWV population as an indicator of colony health.
2. This study uses coordinated treatment of Varroa in a geographically isolated environment (Isle of Arran, Scotland) over 3 years. The study area contained 50–84 colonies managed by ~20 amateur beekeepers. Sampling and virus analysis to assess strain diversity and viral loads were conducted before and after treatments, and changes in population diversity were quantified by sequence analysis.
3. Over the 3 years analysis of the virus population revealed that the dominant DWV variant shifted from Type A to Type B in all apiaries, regardless of mite levels or proximity to other colonies. During this period the number of managed colonies increased by 47% (57–84 colonies), but despite this, we estimate total mite numbers decreased by 58%.
4. *Synthesis and applications*. In this study, the beekeepers in Arran significantly improved the number of colonies they managed, without importing any bees onto the island, indicating that an improved focus on management techniques, through the combination of a coordinated miticide programme and an improved understanding of bee diseases, could yield positive results for bee health and sustainability.

## Graphical abstract

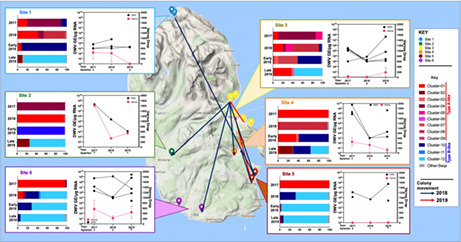

## 1 INTRODUCTION

The detrimental impacts to European honey bee (*Apis mellifera*) colonies of *Varroa destructor* infestations and the viruses they transmit, particularly Deformed wing virus (DWV), are well documented (Dainat et al., 2012; Martin et al., 2012; Tentcheva et al., 2004; Wilfert et al., 2016). Annual colony losses are high, with figures in the US of 37.7% in 2018, 22.2% in 2019 and recently 39% in 2022 (Aurell et al., 2022; Bruckner et al., 2019, 2020) and losses in Scotland of 23.7% and 18.9% in 2017–18 and 2018–19 respectively (Gray et al., 2019, 2020). Despite the availability of a range of miticides, Varroa infestations remain the predominant cause of these reported overwinter colony losses (Dainat et al., 2012). In certain cases, this is due to miticide resistance in the mite population, such as pyrethroid resistance (Thompson et al., 2002). In others, it is due to incorrect or inappropriate miticide application, or the omission of treatment (Jacques et al., 2017). Beekeeping practice therefore has a fundamental impact on colony health. The movement of bees (through the activities of drifting between colonies or robbing weaker colonies) can result in reinfestation of miticide‐treated colonies (Peck & Seeley, 2019), and it is therefore recommended to coordinate treatment with neighbouring hives, and it would seem logical to extend this to include nearby apiaries. However, predominantly independent beekeepers rarely coordinate treatment at the landscape scale and, in the absence of regulation or definitive evidence of the benefits to bees of coordinated treatment, there remains the ongoing risk of reinfestation from nearby unmanaged, mismanaged and feral colonies or even from colonies treated appropriately, but at different times of year.

The dynamics of DWV infections are dramatically altered by the introduction of Varroa. Low‐level highly diverse viral populations that rarely cause symptoms are transmitted between bees by feeding and trophallaxis, with the virus population contained in the gut of healthy bees. The introduction of Varroa changes the transmission route as the mites inject viruses during feeding, leading to high virus titres, causing symptomatic infections and increased mortality (Dainat et al., 2012; Highfield et al., 2009; Martin et al., 2012; Ryabov et al., 2014). Changes to the diversity of the DWV population, as well as the virus titre, are a known marker of changes to colony health, with reductions in DWV population diversity associated with the introduction of mites at the colony level (Martin et al., 2012), and mixed virus populations at the colony level typically associated with low virus/mite levels (Ryabov et al., 2014). However, more recent research has indicated colonies with high virus levels can still have mixed virus populations (Annoscia et al., 2019; Ryabov et al., 2019; Woodford et al., 2022). There are two major variants of DWV, known as Type A and Type B, and studies have indicated there is little difference in terms of the pathogenicity for bees when injected (Gusachenko et al., 2021; Gusachenko, Woodford, Balbirnie‐Cumming, Campbell, et al., 2020; Tehel et al., 2019). However, recent studies have reported a more widespread occurrence of Type B‐like variants during field sampling in the UK, USA and Europe (de Souza et al., 2019; Kevill et al., 2019; Natsopoulou et al., 2017; Ryabov et al., 2017), including when mite levels were low in colonies (Norton et al., 2021). Norton et al (2021) showed that Type B persisted in miticide treated colonies, whilst Type A was correlated with mite levels in the colonies, indicating a potential selective advantage for Type B variants.

Investigation of virus replication in mites has indicated that Type B‐like variants and its recombinants replicate in Varroa, but that Type A‐like variants do not and are likely vectored in a non‐propagative manner (Gisder & Genersch, 2020; Gusachenko, Woodford, Balbirnie‐Cumming, Campbell, et al., 2020; Posada‐Florez et al., 2019). This offers another possible explanation for the recent emergence and spread of Type B‐like variants in surveys and emphasizes the need for improved mite‐control programs. One possible area for improvement is the geographic coordination of treatment.

Coordinated treatment programmes have a long history of use in successful disease control and eradication, whereby management is carried out over a geographically defined area to the benefit of the individuals within that area. These have included cattle tick fever eradication (Pérez de Leon et al., 2012), visceral leishmaniasis control (Boelaert et al., 2000), and Zika virus control in South America (Barrera et al., 2019). One of the most well‐known coordinated treatment programmes in the UK is the treatment of sea‐lice in marine salmon farms. Sea‐lice have free‐living larvae capable of moving between farms using currents and as such coordination of treatments in the fish farms is critical to control the lice (Murray & Salama, 2016). These coordinated programs can take the form of applying a chemical to multiple sites simultaneously, as with the fish farms, or through dissemination of information to the public to improve understanding, as with the mosquito control programs used to reduce the spread of Zika virus (Barrera et al., 2019).

Similar area‐wide coordination programmes can benefit bee health, through appropriate miticide treatments and dissemination of information to amateur beekeepers. Based on 2021 census data, there are approximately 270,000 reported honey bee colonies in the UK, managed by beefarmers and amateur beekeepers (National Bee Unit, 2022). Across much of the UK, these colonies share a contiguous environment with overlapping foraging ranges and widespread interbreeding between colonies, as well as frequent movement of colonies by beekeepers. This environment creates opportunities for interhive transmission of diseases and makes large‐scale disease management difficult. There are few exceptions to this in the UK; for example, colonies kept in remote locations, in valleys, in mountainous areas, or on islands.

There are a limited range of miticides approved for use in the UK (VMD Defra, 2022), and environmental conditions (presence/absence of brood, nectar flow, weather conditions) further restrict the options for effective Varroa control. It therefore makes sense to maximize the efficacy of relevant treatments by controlling Varroa at the landscape scale.

Previous attempts to coordinate the treatments of Varroa infestations of honey bee colonies have been unsuccessful or carried out on an insufficiently large scale (a single apiary) to be informative (Giusti et al., 2016; Sampson & Martin, 1999). Therefore, a thorough long‐term study of coordinated treatments of colonies, across multiple sites in an isolated environment of an appropriate size is required. In this study, we investigated the consequences of coordinated miticide treatment of all known managed colonies in a geographically isolated environment, the Isle of Arran, Scotland. To make coordination more likely to be achieved, we considered factors such as isolation and numbers of beekeepers and hives within the environment. Whilst the ideal might be a single beekeeper occupying an isolated location with a large number of colonies, pragmatic considerations on accessibility and the availability of suitable sites were of paramount importance. Bees cannot fly over large bodies of water (Simpson & von Frisch, 1969), and as Arran is a minimum of ~5 km from mainland Scotland this controls for any drifting or robbing workers or swarms which may carry mites into the treated colonies/apiaries. We studied the resulting level of Varroa infestation and the altered dynamics of the DWV population, both key markers of colony health. We found a significant decrease in mite abundance between the first year of treatment and the third, suggesting that coordinated treatments were reducing *Varroa destructor* infestations in these colonies, and a large increase in the number of managed colonies by the end of the study. Here, we show the first evidence of large scale coordinated treatments for Varroa infestations on honey bee colonies with clear improvements for colony health.

## 2 MATERIALS AND METHODS

### 2.1 Location and beekeepers

This study did not require ethical approval and no permission was required for fieldwork. The island chosen for the study, the Isle of Arran, is off the west coast of Scotland, approximately 5 km from the mainland at any point. It is approximately 167 km^2^ in area and most of the population (~6500) live in small coastal villages. The north of the island, containing one of the beekeeping sites, is divided from the rest of the island by a range of hills over 800 m high.

**FIGURE 1:**
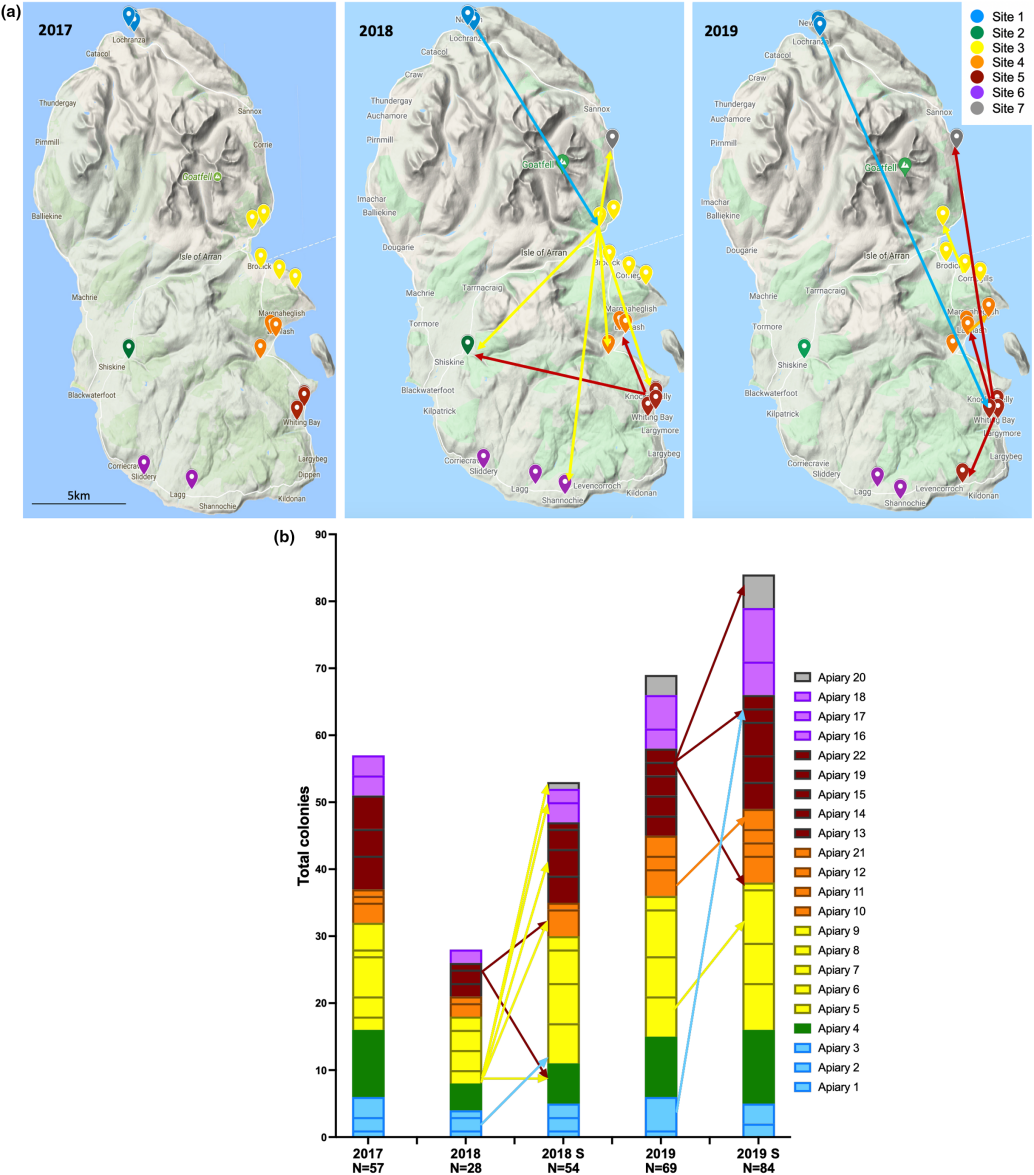
Apiary locations, sites and colony movements throughout study. (a) Apiary locations during the study period on the Isle of Arran. Each marker indicates a single apiary, coloured by site. The arrows on the 2018 and 2019 maps indicate the movement of colonies, coloured by site, from the source to destination apiary. (b) Bar plot indicating the total number of colonies at each sample collection point as a percentage of the total. The same arrows as A are shown, indicating colony movements.

The local beekeeping association, the Arran Bee Group (ABG), agreed to host the study which involved a coordinated late summer miticide treatment and—in a change from their previous practice—the application of a mid‐winter treatment. In 2017, the 17 members of the ABG agreed to stop honey bee imports for the duration of the study. At this time, there were <60 managed colonies on the island distributed between six ‘sites’. Figure 1a shows maps of all the apiaries coloured by ‘site’. These were regions, often containing several apiaries, grouped by their relative distance from one another and centred on the major population areas.

### 2.2 Treatments and sample collection

When available, worker/nurse bees were sampled from all colonies at each spring or summer visit (Table 1) to exclude bees drifting or robbing from adjacent hives. The brood was not sampled to avoid disruption of honey production and damage of brood frames. Bees were stored alive in tubs with fondant until being snap‐frozen and stored at −80°C prior to further analysis.

**TABLE 1:** Site visits, treatments and sample collection on the Isle of Arran. Mite collection was carried out whilst Apivar treatments were still being applied to the colonies.

Year	Honey bee collection	Treatment application (Apivar)	Mite collection	Treatment application (Api‐bioxal)
2017	August	1st week—September	For 7–8 days after treatment applied	December
2018	July	1st week—September		December
2019	May & August	1st week—September		December/January 2020

Two miticide treatments were applied each year, a coordinated late summer application of amitraz (Apivar, Véto‐pharma, Palaiseau, France) to all colonies for a minimum of 6 weeks and a mid‐winter oxalic acid (Api‐bioxal, Véto‐pharma, Palaiseau, France) dribble. Apivar consists of 2 × plastic strips impregnated with amitraz, which were placed in the colonies for a minimum of 6 weeks according to manufacturer's instructions, typically 3 frames in from the edge of the brood box to maximize exposure throughout the brood chamber. Upon Apivar treatment paper collection trays with a mesh grid were placed on the hive floor for 7 to 8 days to allow the dead mites which dropped from the colony to be quantified. The mid‐winter treatment was not coordinated as bees were not foraging, and therefore not drifting/robbing. Api‐Bioxal was applied as a 3.6% w/v solution in 1:1 w/v syrup (granulated sugar and water) as per the manufacturer's instructions. If mite levels are controlled in late summer, sufficiently early to protect the winter bees from virus damage, then residual mites that survive treatment reproduce in late‐season brood. This necessitates the winter miticide application. Table 1 outlines when the various aspects of the project were conducted with precise timings depending upon environmental conditions.

### 2.3 RNA extractions and cDNA synthesis

Frozen honey bees were homogenized individually in year one and in pools of ~30 bees per apiary in all years using liquid nitrogen and a mortar and pestle. RNA extractions were performed using the GeneJET RNA purification kit (Thermo Fisher) as per manufacturer's instructions. RNA was quantified by Nanodrop‐1000 and total cDNA was synthesized from 1 μg RNA per sample using a qScript cDNA synthesis kit (Quanta Biosciences), following the manufacturer's protocol using oligo(dT) and random primers.

### 2.4 PCR and qPCR analysis

For absolute viral load, quantitative polymerase chain reactions (qPCRs) were performed for DWV in a Bio‐Rad C1000 Thermal Cycler (Bio‐Rad). Reactions were set up using 1x Luna Universal qRT‐PCR master mix (New England Biolabs), 0.25 μM forward and reverse primers and 100 ng of cDNA in a final volume of 20 μL. The DWV forward primer was 5′ATATAGGTTCGGCTGGATCTCC 3′ and the reverse primer was 5′TTCCAGATGCACCACACATGC 3′, amplifying a region of 150 bp in the helicase. Amplification was performed using the following thermal profile: 1 min at 95°C, followed by 40 cycles of 15 s at 95°C and 30 s at 60°C. Negative template controls and a serial dilution of a positive control standard were included in each run. DWV genome equivalents were calculated from the standard curve generated from a serial dilution of a cDNA clone control obtained from 1 μg of DWV VVD RNA transcript as per (Gusachenko, Woodford, Balbirnie‐Cumming, Ryabov, et al., 2020), with a linear range of 10^3^–10^10^ GE/μg.

### 2.5 Sequencing and virus population analysis

To investigate whether there were changes in virus diversity during the study period, both over time and between sites, ~10 kb amplicons of the DWV genome were generated using a long‐amp PCR protocol as per (Gusachenko, Woodford, Balbirnie‐Cumming, Ryabov, et al., 2020) from pooled samples of 30 bees for each apiary. These were sequenced and analysed using the NGS analysis software ShoRAH (short read assembly into haplotypes; Zagordi et al., 2011) to generate haplotypes from each sample set. The near‐full length amplicons of the DWV genome were purified and sequenced using an Illumina HiSeq (all reads available under the NCBI BioProject: PRJNA811499). 150 bp paired‐end Illumina reads were trimmed using Geneious Prime (v.2019.1.3) and extracted as a single fasta file for each sample pool. Reads were aligned to the DWV VVD genome (Acc No ‐ MT415950.1) using BWA (Li et al., 2009; Li & Durbin, 2009) and haplotype diversity was calculated for each sample using ShoRAH (Short read assembly into haplotypes), with all settings run as per the default (Zagordi et al., 2011). All haplotypes >2% of the viral population were included in final population diversity analysis, based on a positive control threshold (Woodford et al., 2022).

Single nucleotide variants (SNVs) were called using statistical modelling at a frequency lower than the error rate of sequencing. ShoRAH analysis includes a statistical test of strand bias by utilizising a Fisher's exact test and a Benjamini‐Hochberg correction process, which rejects any *p*‐values ≤0.05 in the final analysis of SNVs. Based on this method, combined with the probabilistic clustering method implemented by ShoRAH, SNVs can be called from deep sequenced viral populations with high accuracy (McElroy et al., 2013). We utilized this method to evaluate the sequence diversity changes observed in colonies over time.

### 2.6 Statistical analyses

Statistical analyses of DWV and mite changes were performed using Graphpad prism (v9). Unpaired, non‐parametric Mann–Whitney tests were used to examine the significance of DWV changes between the years of sample collection. Unpaired *T*‐tests were used to test the significance of changes in *Varroa* drop year to year. To analyse changes in the dominant variant of DWV based on haplotype sequencing a linear regression model was implemented using the lme4 package (Bates et al., 2015) in R studio (v4.0.4) (R Core Team, 2021). The model used DWV as the response variable and examined interactions between the dominant variant and year and the dominant variant and Varroa levels.

## 3 RESULTS

### 3.1 Colony locations and movements

In Figure 1a, the apiaries for each of the 3 years of treatment are displayed, with coloured arrows indicating hive transfers. No restrictions were put in place to prevent intra‐island colony exchange or movement. Figure 1b highlights the change in the total number of colonies managed throughout the study and their distribution. In 2017 there were 17 apiaries with 57 colonies. Over 50% of colonies died by Spring 2018 (29/57) and replacements were moved around the island. Two large apiaries maintained by the ABG at site 3 and 5 supplied most of the replacement stocks via splits and swarm management. Splits were made by rearing new queens and taking a subpopulation of the workers from strong colonies to create new colonies. By the last visit in 2019 the ABG members were managing 84 colonies.

### 3.2 Investigating DWV diversity changes over time

**FIGURE 2:**
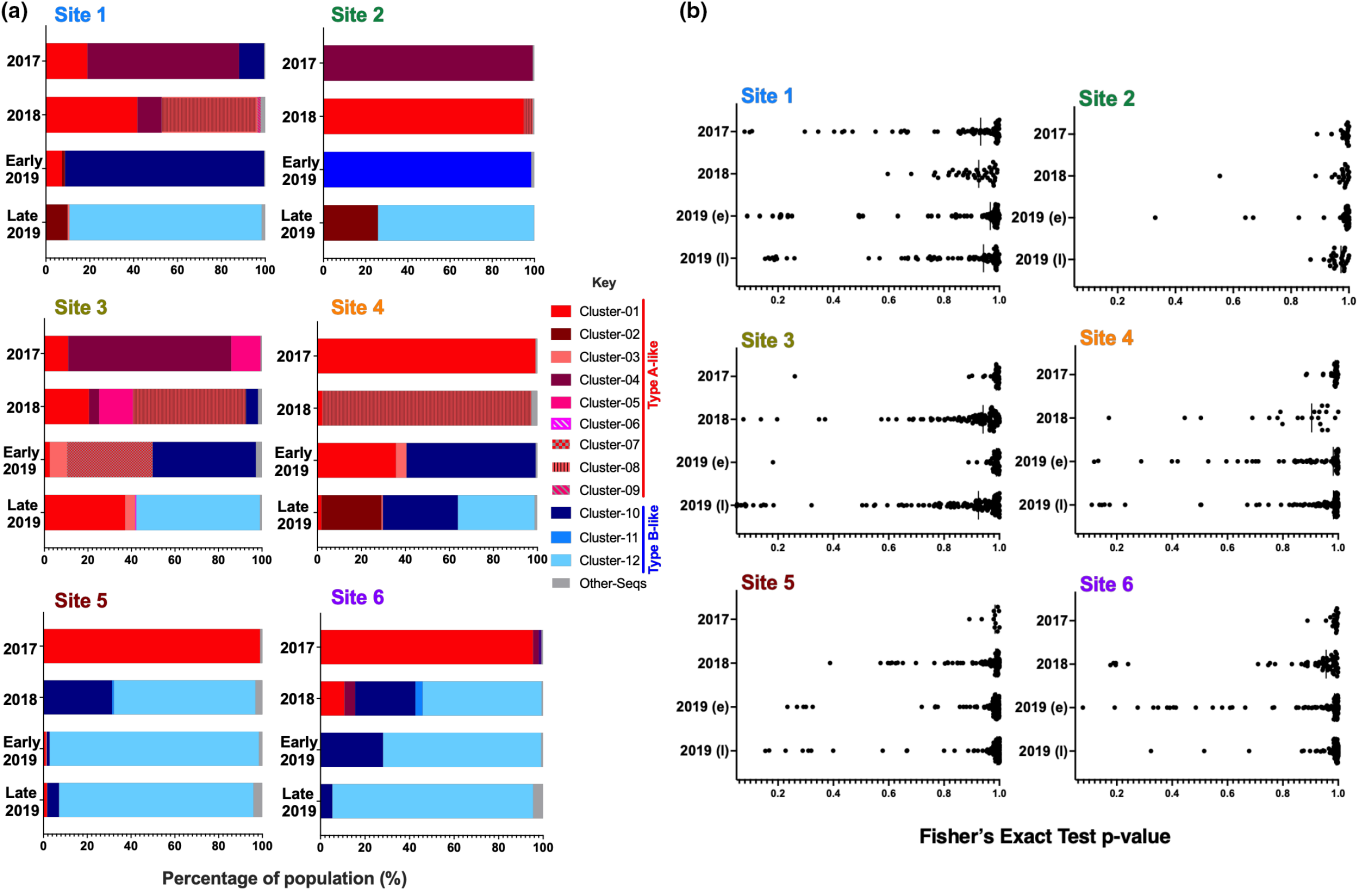
Barplot analysis of DWV haplotype clusters and associated SNV analysis for each site. (a) The assigned clusters from the phylogenetic analysis were used to generate bar plots for each site and year. The clusters were coloured shades of red for Type A like sequences and shades of blue for Type B like sequences. Sequences which fall below the QC limit, defined as 2% in the materials and methods, are classed as ‘other‐seq’. A breakdown of the percentage of each set assigned to each cluster is shown in Table S1. (b) Single nucleotide variants (SNVs) for the RdRp region generated from ShoRAH analysis for all samples from Arran were called if present in 3/3 iterations of the modelling. SNV *p*‐values close to 1.0 represent a variant in the majority of sequences in that dataset differing from the reference sequence. Samples with SNVs with lower *p*‐value scores therefore have a greater amount of sequence variation within the sample. Anything with a *p*‐value ≤ 0.05 was excluded as the threshold for error based on McElroy et al. (2013) modelling data.

The sequence output from ShoRAH was used to generate neighbour‐joining phylogenetic trees to determine population diversity amongst the different sites. Sequences were assigned to ‘clusters’ based on their clade alignment (Figure S1). The resulting clusters were plotted by site and year to reveal changes over time (Figure 2a). A shift from DWV Type A‐like sequence dominance in 2017 to DWV Type B‐like dominance in 2019 was observed across all six sites. Sites 1–5 contained a mix of Type A‐like and Type B‐like sequences in 2019, but Type B was dominant in all by the end of sampling and was the only virus present in the site 6 samples.

Analysis of the SNVs (single nucleotide variants) generated from the ShoRAH analysis (McElroy et al., 2013) was also performed to confirm the haplotype diversity (Figure 2b). The results validate the phylogenetic analysis and cluster assignments, with little diversity observed in 2017, except for site 1, increasing diversity in 2018 across some sites as Varroa and DWV levels decreased, and some diversity in both 2019 sampling points. The SNV analysis also confirmed some of the site‐to‐site variation, with site 2 showing little diversity, whilst sites 3 and 4 had high SNV diversity in 2018, in agreement with the cluster analysis.

A multiple linear regression model with interactions was used to examine the variables that may have influenced DWV levels during the study. It was observed that clonality in a sample and a decrease in the number of haplotypes observed correlated with increased Varroa levels and DWV titres in the colonies throughout the study (Figure S2). No two‐way interactions were significant by this analysis method. The results of the NGS analysis suggest that there is a landscape shift from Type A to Type B DWV variants occurring regardless of the proximity of the colonies to one another or the titre of DWV observed during sampling.

### 3.3 Treatments, Varroa drop and DWV titre

A good indicator of colony health and mite infestation levels is the DWV titre observed in the bee population, with high virus titres typically associated with mite infestations, symptomatic virus infections (>5 × 10^6^ GE/μg RNA; Gusachenko, Woodford, Balbirnie‐Cumming, Campbell, et al., 2020) and colony losses (Dainat et al., 2012; Highfield et al., 2009). To measure changes in the virus titre in colonies, nurse bees were collected annually prior to each miticide application, typically in late summer (Table 1).

**FIGURE 3:**
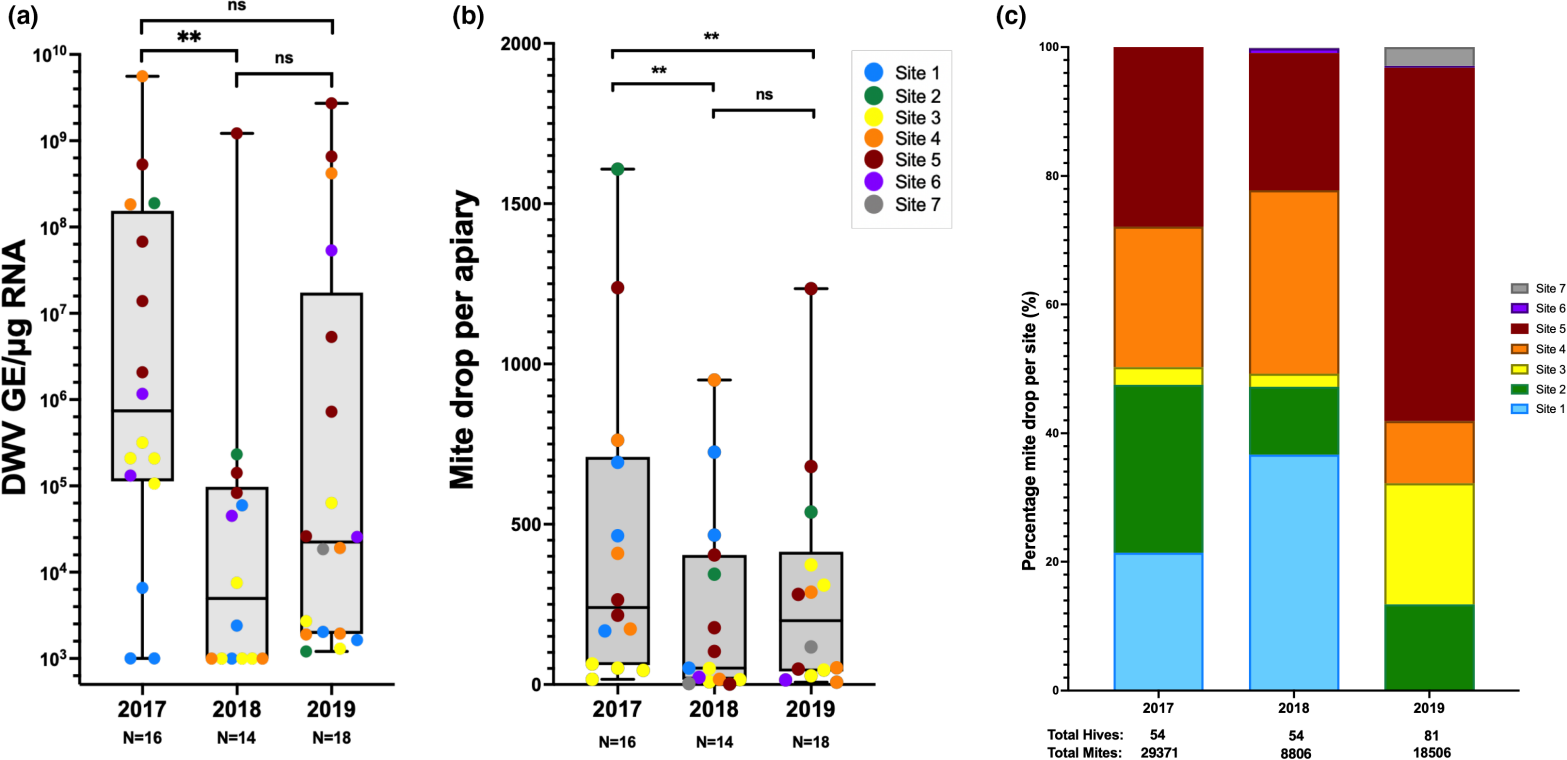
Pooled apiary DWV and individual colony mite drops and the change in proportion of mite drop between sites. (a) each point shows the quantified viral load for a single apiary on the island, the points are coloured by location and split per year of sampling. (b) Each point shows the average mite drop for an apiary at each of the seven sites in each of the 3 years of treatment. The *n* = X below each year indicates the number of apiaries analysed. The significance values shown at the top are p‐values determined using a Mann–Whitney non‐parametric test. (c) Year on year contribution of sites to the total Varroa drop across all sites after 1 week of Apivar treatment. Each coloured block represents the drop for all the apiaries in that site as a percentage of the total mite drop. Where sites are not shown it indicates no mites were recorded post‐treatment in those apiaries. The numbers below the *x*‐axis indicate the number of colonies treated and the total mite drop. Note that hive numbers differ from sample points in Figure 1b, as colony numbers changed between sample collection and treatment application.

Pools of worker/nurse bees (~30 bees) were analysed by qPCR and compared to examine changes in DWV titre over time. Figure 3a shows the changes in DWV titre between each year with each apiary coloured by the site it is located in. The average DWV titre significantly decreased between 2017 and 2018 from 8 × 10^5^ GE/μg RNA to 6 × 10^3^ GE/μg RNA (Mann–Whitney *p*‐value ≤0.01), but was not significantly different between 2017 and 2019, despite the average in 2019 only increasing to 4 × 10^4^ GE/μg RNA. Two sites (site 2 and 5) had consistently higher virus titres than others throughout the duration of the study and these broadly correlated with higher mite levels (Figure 3b). Site 1 had low DWV titres in each year, despite having high mite levels in 2017 and 2018, but other sites with low DWV titres, typically observed in healthy colonies, correlated with lower Varroa levels (site 3, site 6).

When Apivar was applied to each colony in September (Table 1), paper trays were placed on the floors of the hives to collect the dead mites which fell from the bees/frames in the first 7/8 days of treatment application (Titera & Haklova, 2003). Figure 3b shows the average mite drop for each year with each data point representing an apiary average. In 2018 and 2019, most colonies had <1000 mites, a number not requiring immediate intervention according to the UK's National Bee Unit Varroa management recommendations (National Bee Unit, 2022), with 1 colony in 2018 and 4 colonies in 2019 containing >1000 mites, compared with 10 in 2017. Using an unpaired t‐test, the statistical significance between each year was calculated, with changes from 2017 to 2018 (*p*‐value 0.0012) and 2017 to 2019 (*p*‐value 0.0027) found to be statistically significant, but changes between 2018 and 2019 not (*p*‐value 0.35; Table S2). The average mite‐drop per colony decreased by 55.6% between 2017 and 2019, as shown in Table 2. The mite drop for each apiary was calculated as a proportion of the total drop on the island for each year to examine site‐to‐site differences over time (Figure 3c). Mite levels were relatively evenly distributed between sites 1, 2, 4 and 5 in 2017, but by 2019 > 50% of mites counted were found in site 5 colonies.

**TABLE 2:** Changes in mite abundance and average DWV titre over time. The average DWV titre shown is per apiary, rather than per colony, as these were analysed in pools. The change in mites is calculated as a percentage change from in the annual average (compared to the 2017 pre‐treatment). All colonies which recorded zero mites in a drop were excluded from analysis.

Year	Total colonies	Total apiaries	Average DWV per apiary (GE/μg RNA)	Total mites	Average per colony	Change in mites from 2017
2017	43	17	8 × 10^5^	29,371	683.0	/
2018	39	16	6 × 10^3^	8806	225.8	67% decrease
2019	61	19	4 × 10^4^	18,506	303.3	55.6% decrease

**FIGURE 4:**
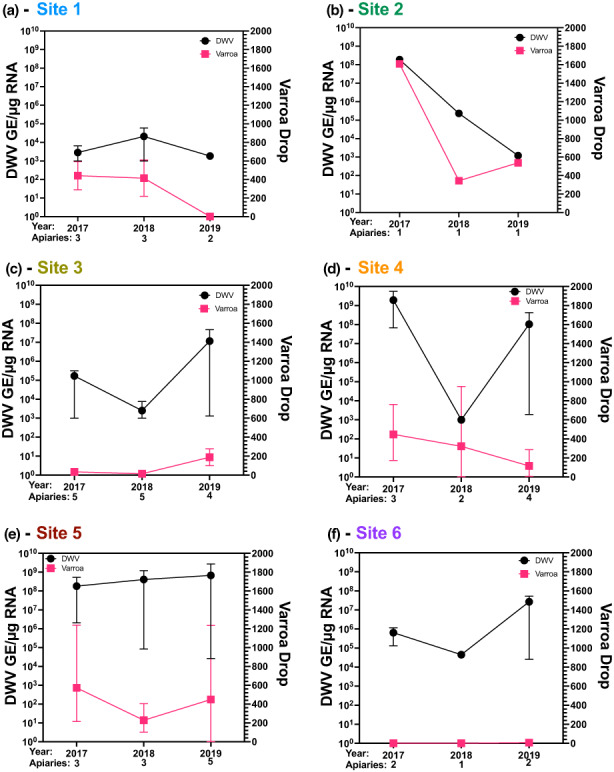
Site by site DWV and mite drop averages across 3 years. (a–f) Viral loads for pooled samples of site 1–6 from 2017, 2018 and 2019 shown per site and the average mite drop shown per region. Each black dot represents the DWV titre from one apiary and the pink squares represent the average Varroa drop for the whole site. The number of apiaries at each time point are shown along the *x*‐axis. The error bars represent the average apiary range for each sample set. Site 7 is not shown as it only became an apiary and joined the study in late 2018.

To compare the differences in virus titre and mite drop observed across the island, the changes were plotted per site (Figure 4). The plots reveal changes on a site‐by‐site basis, with an observed reduction over time in mite abundance at sites 1, 2 and 4 together with reducing or steady DWV titres over time. Sites 3, 5 and 6 remain largely unchanged or showed slightly increased mite and DWV levels. This highlights that the changes to the markers of colony health are occurring on a site‐by‐site basis as well as at an island level, perhaps reflecting the fractured distribution of colonies on the island. The year‐to‐year differences observed are likely to be influenced by colony movements between sites and external variables like the temperature each winter, with harsher winter conditions more likely to result in the demise of a colony already burdened with high levels of mites and viruses.

### 3.4 Overwintering losses in year one

In the first year of the study, worker/nurse bees were analysed individually by qPCR and the average DWV titre from 5 bees per colony was plotted against the Varroa drop to correlate the association between the two and colony deaths (Figure S3). The points with hollow circles (26/54) indicate which colonies died over the winter of 17/18. Met office data for the island indicated a notably harsh winter between November 2017 and March 2018, which will have impacted the development of the bees the following spring (Figure S4 and Table S3). Anomaly data indicated that November and February were 1.5°C cooler and March was 2.5°C cooler than the 30‐year average, emphasizing the harsh conditions of the 2017/18 winter (Met Office, 2022).

## 4 DISCUSSION

The use of coordinated treatments across large geographical areas to reduce or prevent the transmission of infectious diseases has been effective for a range of human and animal pathogens, including both Zika virus and Visceral Leishmaniasis control, cattle tick fever eradication and sea‐lice infestation of salmon farms (Barrera et al., 2019; Boelaert et al., 2000; Murray & Salama, 2016; Pérez de Leon et al., 2012). In this study, we used an island as a geographically isolated environment to carry out a coordinated treatment of honey bee colonies with miticides to reduce *Varroa destructor* infestations. Varroa is the major pathogen of European honey bees and, if unmanaged, causes excessive over‐wintering colony losses. An example of the devastation Varroa can cause is evidenced by studies on the island of Gotland in Sweden, where mite‐infested untreated colonies declined in number from 150 to 11 in 5 years as the researchers attempted to select for natural mite resistance (Fries et al., 2006). It is further emphasized by the successful use of deliberate Varroa infestation to eradicate introduced honey bee colonies on Santa Cruz Island, California (Wenner et al., 2009).

There are a wide range of miticides available to control Varroa and many of these commercial products recommend application in a coordinated manner. Despite this, there is little to no evidence that beekeepers—>95% of which are small‐scale hobbyists in the UK—apply treatments in a coordinated manner. In the UK, there is no regulatory requirement to treat Varroa infestations, whether coordinately or not. Colonies with low mite levels, located near highly infested colonies, rapidly acquire mites and the level of DWV in the individual mite‐infested bees increases exponentially (Woodford et al., 2022), often resulting in the colonies dying over winter. To investigate the potential benefits and practical problems of coordinate treating at the landscape scale we worked with the Arran Bee Group, an engaged beekeeping association that had previously experienced unsustainable colony losses almost certainly due to Varroa infestations. The island location provided a geographically isolated area in which to conduct the study. The Arran Bee Group, to which all the known beekeepers on the island belong, agreed to bring no imported colonies onto the island during the study period to prevent the possible infestation of established colonies with mites or altering the virus diversity present. Instead, increasing or replacing colonies was achieved by splitting existing healthy colonies and rearing new queens, with many of these colonies moved for the purposes of restocking after winter losses, apiary expansion or the provision of bees to new beekeepers.

The coordinated miticide treatments across 3 years on Arran showed improvements in overall mite levels (55.6% overall reduction between 2017 and 2019 based on numbers collected using a standardized method, Table 2) and on the number of colonies managed on the island (up from 54 to 81 between 2017 and 2019, Table 2). An increase in the number of colonies managed indicates an improvement in overall health of the bees on the island, as fewer are dying, and an improvement in beekeeping ability (through splitting of colonies and improved understanding of disease management). Isolated sites, such as site 1 located in the north of the island (Figure 1), reported no mites by the end of the study (Figure 3), whilst other sites (site 2) reported large decreases in mite and virus levels across the 3 years (Figure 4). Several colonies in each year reported no mites post‐drop, however these were removed from all data analyses due to suspected sampling or handling error during mite collection. There was also evidence that colony movement within the island, particularly from site 5, resulted in the continued transmission of mites from one apiary to another, and exacerbated mite and virus numbers at the landscape level. It was interesting to observe that site 1 reported no mites throughout the remainder of the study, likely due to the relative isolation from other colonies on the island (Figure 1a). This, and the continued infestation of colonies in the more densely populated regions of the island (site 5), indicate that colony proximity is one of the most significant factors for mite management, regardless of coordinated treatments, and would influence any future studies into landscape mite management in the UK or elsewhere.

In the first year of the study, the beekeepers lost a large number of colonies (29/54) over winter and these losses broadly correlated with high mite and DWV levels (Figure S3). This occurred despite the application of the first coordinated treatment and is most likely explained by an already very high level of mites in the colonies (2000+ mites were recorded from individual colonies during the first treatment regime) resulting in weakened colonies overwintering. This was coupled with a notably harsh winter between December 2017 and March 2018 (2–3°C cooler than the 30‐year average) which will have impacted the development of the bees the following spring (Figure S4 and Table S3). Previous studies have suggested that low mite numbers do not necessarily translate to low DWV levels and colonies could still collapse in the winter, despite treatment application (Highfield et al., 2009). In this study, no colonies were reported to have very high DWV levels with low mite numbers (Figure S3), and our recent studies have clearly demonstrated that effective miticide treatment of colonies with high Varroa and virus levels resulted in massive reductions of the viral load within 4 weeks (Woodford et al., 2022). Interestingly the average titre of DWV across the island did not significantly decrease between 2017 and 2019, despite the drop in average mite levels (Figure 3a and Figure 4). These differences could be explained by the sampling methods, whereby nurse/worker bees were sampled rather than brood samples for practical purposes as discussed in the methods. Sampling workers/nurse bees may mask the true DWV titre in the colony, as highly infected brood may die during development or emerge with deformed wings and quickly perish or be removed from the colony. Despite the average titres not being significantly different between 2017 and 2019, the sites with higher DWV typically had higher mite levels too (site 4 and 5), indicating a correlation between high DWV and high Varroa (Dainat et al., 2012; Highfield et al., 2009).

The DWV population at all six sites underwent a dynamic shift from Type A variant dominance to Type B dominance during the 3 years based on sequence analysis (Figure 2). This shift in virus dominance has been reported at a landscape scale in multiple studies recently, including in the UK and the USA, suggesting that this is not a phenomenon associated with coordinated mite treatment (Kevill et al., 2019; Paxton et al., 2022; Ryabov et al., 2017). Experiments run in parallel with this study showed that mite infested colonies contained mixed populations of viruses, but that Type B variants were increasing over time (Woodford et al., 2022). A general shift from Type A to Type B has been observed across the UK (Kevill et al., 2021) and the findings from both this study and Woodford et al. (2022) support those findings. Interestingly the mites sequenced by Woodford et al. (2022) contained mixed populations of DWV, suggesting other selective advantages might be responsible for the increase in Type B occurrence in both that study and this one. Norton et al. (2021) observed that DWV Type B levels persisted in miticide treated colonies, whilst Type A variants were largely undetected in the absence of Varroa, indicating a mite dependency for sustained transmission of that variant. By increasing and decreasing the level of mites in the colonies they were able to demonstrate a positive correlation between higher mite levels in the colony and Type A titres. These studies offer at least a partial explanation for the dynamic shift observed on Arran, where very high mite levels in year one correlate with more Type A virus by sequence analysis, and as the colonies are treated across time more Type B sequences were detected (Figure 2a).

Low levels of Type B were observed at site 1 in the first year and colonies from this location were used to supplement losses at site 3 in year one (Figure 1), which were then also used to split and move colonies further around the island after the winter losses in year one. These movements likely explain, at least partially, the subsequent observation of Type B variants at the other sites on the island. The bee group were unaware of any established feral colonies, but potential infestation of treated colonies from robbing or drifting workers from swarms or unknown feral colonies may also have influenced the dynamic shift in the virus population, with mites introducing new variants during feeding. However, since the shifts in the virus population broadly correlate with the movement of colonies around the island and there were no ‘unusual’ virus variants observed in the sequencing studies that may have arisen from long‐established feral colonies we favour the former reason probably accounts for the eventual dominance of type B viruses. This may have occurred through a stochastic process leading to Type B dominance through chance (Woodford & Evans, 2020) or it may be because colonies infected with Type B are actually more tolerant to infection, and therefore stronger and more likely to be split by the beekeepers, than those with high levels of Type A.

The considerable cooperation of the beekeepers on which this study relied depended upon minimal disruption to managed colonies, which included not harvesting sealed brood. Therefore, all bees sampled on the island were young adults taken from the comb. It is possible that the brood parasitized by the mites would carry other variants of DWV which are more ‘virulent’ and kill the developing pupae, and therefore not observed in the workers sampled in this study. Additionally, as the sequencing analysis was generated using large amplicons (10 KB + fragments), it is possible Type B variants were below the threshold of detection at some sites in the first season. Regardless, the shift from one variant to the other across time and space is striking and indicates a potential selective advantage for Type B like variants.

Despite coordinating the treatments for 3 years, mites persisted across most apiaries and some of the locations still had very high mite levels normally associated with subsequent winter colony losses. The study design involved the application of achievable practical beekeeping control measures rather than rigorous interventions to minimize mite numbers—and consequently DWV levels—in the colony. The latter is achievable using more involved interventions; we have recently demonstrated that the combination of a shook swarm (involving safely transporting the queen, then shaking all adult workers into a new colony, before destroying all of the sealed, and potentially mite‐infested, brood) and Apivar treatment can reduce DWV levels by an average of 10^6^ GE/μg in adult workers in the hive within one month (Woodford et al., 2022). Other factors, beside the coordination of the treatments, will influence the mite numbers in the colony, including the rate of colony expansion in spring (Martin, 2001), the proportion of drone comb in the colony and the mite infestation levels in nearby hives and feral colonies (Peck & Seeley, 2019; Thompson et al., 2002). Despite our best attempts to control all colonies on the island, feral colonies or swarms could have cohabited the space and acted as sources of reinfestation via robbing or drifting workers, carrying phoretic mites into treated colonies (Peck & Seeley, 2019; Thompson et al., 2002). Nevertheless, the numbers of colonies managed by the Arran Bee Group increased and the average mite levels per colony decreased over the study period. So, despite not eradicating mites on the island, there was some success from the coordinated treatment regime resulting in an overall improvement in colony health and management.

There are a limited range of miticides approved for use in the UK (VMD Defra, 2022), and environmental conditions (presence/absence of brood, nectar flow, weather conditions) further restrict the options for effective Varroa control. It therefore makes sense to maximize the efficacy of relevant treatments by controlling Varroa at the landscape scale. At a minimum, this should be within an apiary (particularly if shared with other beekeepers) together with the immediate environment, up to a distance over which bees might drift, or robbing can occur. Hive density over much of the UK is very high (<0.5–>3.5 apiaries/km^2^ based upon figures from the National Bee Unit (National Bee Unit, 2022), with which the majority of beekeepers are registered) meaning that coordination over larger areas (possibly to encompass all colonies within a beekeeping association) would be necessary for effective mite control. Since such areas share broadly similar environmental conditions—climate, weather and nectar availability—and are likely to be covered by a local beekeeping group, it would be logical and beneficial to bee health to promote coordination via national and local beekeeping associations. Additionally, we believe consideration of the following points would aid the translation of this work into other settings: raising awareness amongst beekeepers of the benefits of coordinated treatments due to drifting and robbing bees; creating a clearly defined boundary for treatment groups, typically an association, which will likely have geographical boundaries; consideration of climate and forage, for example an area covering lowland and mountain sites is unlikely to be controlled in a single day. Taking these factors into account, we believe coordination of miticide treatments can benefit honey bee colonies in the UK and further afield.

## AUTHOR CONTRIBUTIONS

Luke Woodford, Fiona Highet and David J. Evans conceived the ideas and designed methodology; Luke Woodford, Graeme Sharpe, Fiona Highet and David J. Evans carried out fieldwork and collected the data; Luke Woodford analysed the data; Luke Woodford and David J. Evans led the writing of the manuscript. All authors contributed critically to the drafts and gave final approval for publication.

## CONFLICT OF INTEREST STATEMENT

The authors declare no conflict of interest.

## Supporting information


**Figure S1.** Phylogenetic analysis and cluster assignment of ShoRAH generated haplotypes. The sequences from ShoRAH for the RdRp region of the DWV genome were aligned with DWV infectious clones (NCBI accession numbers ‐ MT415950, MT415949 and MT415952) and full‐length Type‐A and Type‐B reference genomes from the NCBI database, labelled with their accession numbers. Each haplotype label indicates the year, the apiary sample number and the percentage of the population that the haplotype represents. Clusters (Clu) are assigned based on sequence similarity. The red and blue bars indicate samples clustering with Type A and B reference genomes respectively. The analysis uses a neighbour‐joining tree with a Tamura‐Nei model (Tamura and Nei, 1993) and 1000 bootstrap iterations to compile. The DWV type C sequence is used as the outgroup for model generation.
**Figure S2.** GLMM analysis of two‐way interactions between variables determining virus diversity. Using haplotypes (A) and clonality (B) as the significant variable in a multiple linear regression model. A single haplotype determined by ShoRAH analysis and Clonality (1) correlates positively with increasing DWV and mite levels. The greater the number of haplotypes observed in a virus population the weaker the interaction becomes, indicating a high number of haplotypes in a sample is associated with lower DWV and mite levels.
**Figure S3.** DWV qPCR titre vs varroa mite infestation in year one. Each circle represents the average DWV level by qPCR analysis from five individually analysed bees from a single colony, coloured by site. Full circles indicate colonies which survived the 17/18 winter, hollow circles indicate colonies which died over that winter. The majority of colonies which died over winter had 100+ mites in their drop and/or >10^6^ GE/μg RNA of DWV in the autumn sampling. Four colonies at Site 6 (purple) died through suspected starvation, queen failures or natural disaster such as storm damage.
**Figure S4.** Daily mean temperature (0900‐0900) (°C) for the isle of Arran between May 2017 and October 2019. Daily temperatures are shown as individual points in each month and coloured by year, the line indicates the average for each month connected and coloured by year. The raw data was provided by the Met Office and was collected from the nearest weather station to Arran – Machrihanish, Latitude 55:44N Longitude 05:70W, altitude 10m. The average temperature between November 2017 and April 2018 was colder than between the same period in 2018‐19.
**Table S1.** Assigned haplotype clusters for each Site. All clusters were assigned based on the clades generated during the phylogenetic analysis shown in Figure S1. The data presented here was used to compile figure 4A. The red clusters are Type A ‐like variants, blue are Type B ‐like and grey are for the remaining % of samples below the thresholds defined for ShoRAH.
**Table S2.** Summary of the unpaired t‐test analysis of mite drop differences between colonies in each year of treatment. The differences in mite drop between 2017‐18 and 2017‐19 were significant, but not between 2018‐19.
**Table S3.** Monthly mean temperature and standard deviation of the mean for the isle of Arran between May 2017 and October 2019. Years are coloured to match the data plotted in Figure 4. The raw data was provided by the Met Office and was collected from the nearest weather station to Arran – Machrihanish, Latitude 55:44N Longitude 05:70W, altitude 10m.

## Data Availability

All NGS data files used in this paper are publicly available in the SRA (short read archive) of NCBI, which is accessible under BioProject ID: PRJNA811499. Weblink— https://www.ncbi.nlm.nih.gov/bioproject/PRJNA811499.
