# Supplementary material for: All together now: Geographically coordinated miticide treatment benefits honey bee health
Source: J Appl Ecol. 2023 Jan 26;60(5):790–802. doi: 10.1111/1365-2664.14367 (PMC10947427; doi:10.1111/1365-2664.14367)
Supplement: Supplementary file 1 — Figure S1. Phylogenetic analysis and cluster assignment of ShoRAH generated haplotypes. The sequences from ShoRAH for the RdRp region of the DWV genome were aligned with DWV infectious clones (NCBI accession numbers ‐ MT415950, MT415949 and MT415952) and full‐length Type‐A and Type‐B reference genomes from the NCBI database, labelled with their accession numbers. Each haplotype label indicates the year, the apiary sample number and the percentage of the population that the haplotype represents. Clusters (Clu) are assigned based on sequence similarity. The red and blue bars indicate samples clustering with Type A and B reference genomes respectively. The analysis uses a neighbour‐joining tree with a Tamura‐Nei model (Tamura and Nei, 1993) and 1000 bootstrap iterations to compile. The DWV type C sequence is used as the outgroup for model generation. Figure S2. GLMM analysis of two‐way interactions between variables determining virus diversity. Using haplotypes (A) and clonality (B) as the significant variable in a multiple linear regression model. A single haplotype determined by ShoRAH analysis and Clonality (1) correlates positively with increasing DWV and mite levels. The greater the number of haplotypes observed in a virus population the weaker the interaction becomes, indicating a high number of haplotypes in a sample is associated with lower DWV and mite levels. Figure S3. DWV qPCR titre vs varroa mite infestation in year one. Each circle represents the average DWV level by qPCR analysis from five individually analysed bees from a single colony, coloured by site. Full circles indicate colonies which survived the 17/18 winter, hollow circles indicate colonies which died over that winter. The majority of colonies which died over winter had 100+ mites in their drop and/or >106 GE/μg RNA of DWV in the autumn sampling. Four colonies at Site 6 (purple) died through suspected starvation, queen failures or natural disaster such as storm damage. Figure S4. Daily mean tem [file JPE-60-790-s001.docx]

**All together now: geographically coordinated miticide treatment benefits honey bee health - Supplementary Files**

**Figure S1 – *Phylogenetic analysis and cluster assignment of ShoRAH generated haplotypes.*** *The sequences from ShoRAH for the RdRp region of the DWV genome were aligned with DWV infectious clones (NCBI accession numbers - MT415950, MT415949 and MT415952) and full-length Type-A and Type-B reference genomes from the NCBI database, labelled with their accession numbers.* *Each haplotype label indicates the year, the apiary sample number and the percentage of the population that the haplotype represents. Clusters (Clu) are assigned based on sequence similarity. The red and blue bars indicate samples clustering with Type A and B reference genomes respectively. The analysis uses a neighbour-joining tree with a Tamura-Nei model (Tamura and Nei, 1993) and 1000 bootstrap iterations to compile. The DWV type C sequence is used as the outgroup for model generation.*


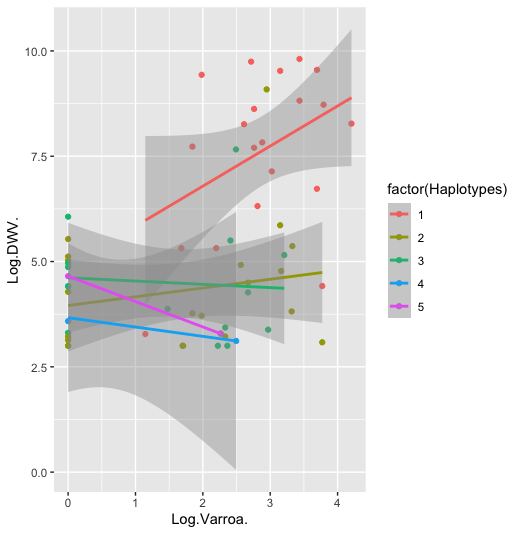

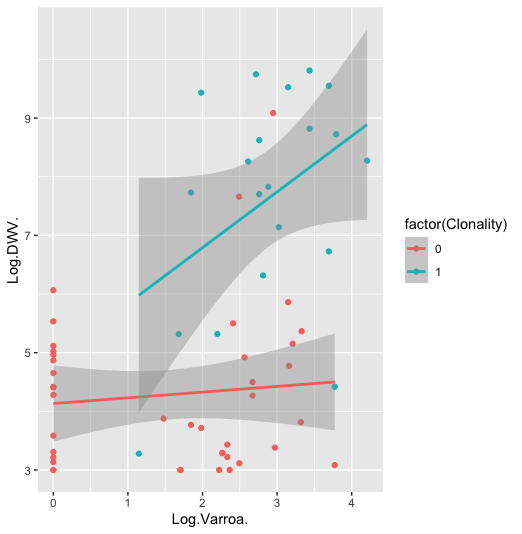


***Figure S2 – GLMM analysis of two-way interactions between variables determining virus diversity.*** *Using haplotypes (A) and clonality (B) as the significant variable in a multiple linear regression model. A single haplotype determined by ShoRAH analysis and Clonality (1) correlates positively with increasing DWV and mite levels. The greater the number of haplotypes observed in a virus population the weaker the interaction becomes, indicating a high number of haplotypes in a sample is associated with lower DWV and mite levels.*


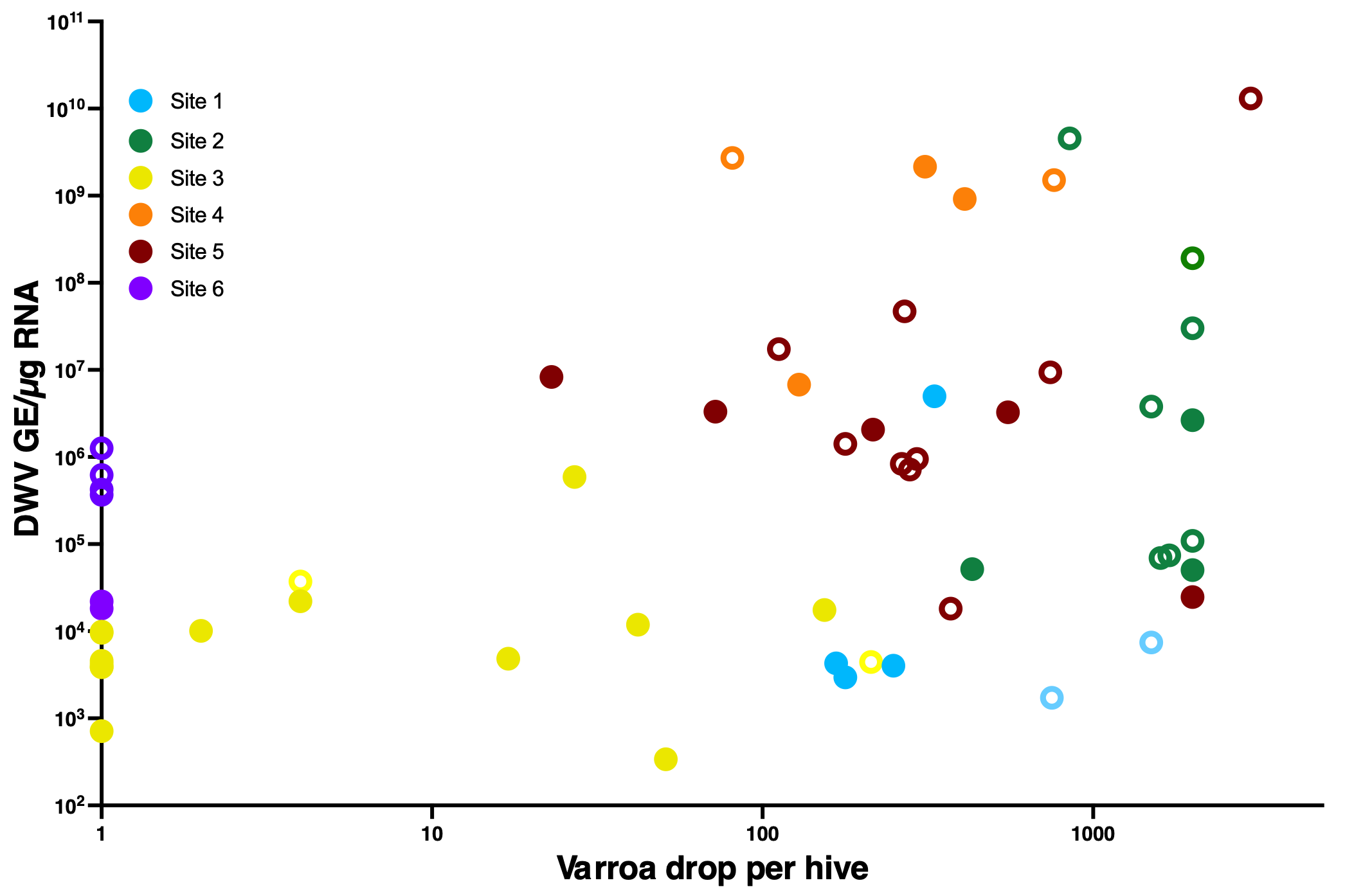


***Figure S3*** ***– DWV qPCR titre vs varroa mite infestation in year one****. Each circle represents the average DWV level by qPCR analysis from five individually analysed bees from a single colony, coloured by site. Full circles indicate colonies which survived the 17/18 winter, hollow circles indicate colonies which died over that winter. The majority of colonies which died over winter had 100+ mites in their drop and/or >10^6^ GE/µg RNA of DWV in the autumn sampling. Four colonies at Site 6 (purple) died through suspected starvation, queen failures or natural disaster such as storm damage.*

***Figure S4 – Daily mean temperature (0900-0900) (°C) for the isle of Arran between May 2017 and October 2019****. Daily temperatures are shown as individual points in each month and coloured by year, the line indicates the average for each month connected and coloured by year. The raw data was provided by the Met Office and was collected from the nearest weather station to Arran – Machrihanish, Latitude 55:44N Longitude 05:70W, altitude 10m. The average temperature between November 2017 and April 2018 was colder than between the same period in 2018-19.*

***Table S1 – Assigned haplotype clusters for each Site.*** *All clusters were assigned based on the clades generated during the phylogenetic analysis shown in Figure S1. The data presented here was used to compile figure 4A. The red clusters are Type A -like variants, blue are Type B -like and grey are for the remaining % of samples below the thresholds defined for ShoRAH.*

**

***Table S2 –*** *Summary of the unpaired t-test analysis of mite drop differences between colonies in each year of treatment. The differences in mite drop between 2017-18 and 2017-19 were significant, but not between 2018-19.*

| **Year** | **R^2^** | **F value** | **P value** |
| --- | --- | --- | --- |
| **2017-18** | 0.12 | 6.873 | 0.0012 |
| **2017-19** | 0.085 | 3.102 | 0.0027 |
| **2018-19** | 0.009 | 2.248 | 0.3488 |

***Table S3 – Monthly mean temperature and standard deviation of the mean for the isle of Arran between May 2017 and October 2019.*** *Years are coloured to match the data plotted in Figure 4. The raw data was provided by the Met Office and was collected from the nearest weather station to Arran – Machrihanish, Latitude 55:44N Longitude 05:70W, altitude 10m.*

|  | **2017** | | **2018** | | **2019** | |
| --- | --- | --- | --- | --- | --- | --- |
|  | **Mean (°C)** | **stdev** | **Mean (°C)** | **stdev** | **Mean (°C)** | **stdev** |
| **Jan** |  |  | 5.0 | 2.18 | 5.5 | 2.73 |
| **Feb** |  |  | 4.0 | 1.99 | 7.1 | 2.85 |
| **Mar** |  |  | 4.4 | 2.09 | 7.4 | 1.94 |
| **Apr** |  |  | 8.0 | 2.39 | 9.3 | 3.02 |
| **May** | 12.0 | 2.45 | 11.5 | 3.02 | 9.9 | 2.41 |
| **Jun** | 13.5 | 1.59 | 13.6 | 2.03 | 13.0 | 2.25 |
| **Jul** | 14.2 | 1.53 | 15.2 | 1.43 | 15.0 | 2.11 |
| **Aug** | 14.3 | 1.21 | 14.1 | 1.51 | 15.3 | 1.81 |
| **Sep** | 12.7 | 1.70 | 12.5 | 1.95 | 13.5 | 1.53 |
| **Oct** | 11.9 | 1.60 | 10.2 | 3.19 | 9.3 | 2.51 |
| **Nov** | 7.3 | 2.04 | 8.3 | 2.30 |  |  |
| **Dec** | 5.9 | 2.95 | 7.0 | 1.76 |  |  |
